# Effective cesium removal from Cs-containing water using chemically activated opaline mudstone mainly composed of opal-cristobalite/tridymite (opal-CT)

**DOI:** 10.1038/s41598-021-94832-y

**Published:** 2021-07-28

**Authors:** Sunki Kwon, Yumi Kim, Yul Roh

**Affiliations:** Department of Earth and Environmental Sciences, Chonnam National University, 77, Yongbong-ro, Buk-gu, Gwangju, 61186 Republic of Korea

**Keywords:** Environmental sciences, Materials science

## Abstract

Opaline mudstone (OM) composed of opal-CT (SiO_2_·*n*H_2_O) has high potential use as a cesium (Cs) adsorbent, due to its high specific surface area (SSA). The objective of this study was to investigate the Cs adsorption capacity of chemically activated OM and the adsorption mechanism based on its physico-chemical properties. We used acid- and base-activation methods for the surface modification of OM. Both acid- and base- activations highly increased the specific surface area (SSA) of OM, however, the base-activation decreased the zeta potential value more (− 16.67 mV), compared to the effects of acid-activation (− 6.60 mV) or non-activation method (− 6.66 mV). Base-activated OM showed higher Cs adsorption capacity (32.14 mg/g) than the others (acid: 12.22 mg/g, non: 15.47 mg/g). These results indicate that base-activation generates pH-dependent negative charge, which facilitates Cs adsorption via electrostatic attraction. In terms of the dynamic atomic behavior, Cs cation adsorbed on the OM mainly exist in the form of inner-sphere complexes (IS) containing minor amounts of water molecules. Consequently, the OM can be used as an effective Cs adsorbent via base-activation as an economical and simple modification method.

## Introduction

The removal of radioactive Cs (^137^Cs) from the Cs-containing wastewater is an important environmental issue, following the nuclear power plant accident at Fukushima Daiichi in Japan. ^137^Cs has a half-life of about 30.2 years with high solubility and mobility rates in water, and due to its chemical properties being similar to those of potassium (K), it can accumulate in plants and animals, increasing the health risk to humans^1–3^. Actual high-radioactive waste seawater shows ^137^Cs to be present in low concentrations (≤ 1 mg/L)^4,5^. Therefore, various studies are under way to develop efficient adsorbents with high selectivity for Cs.

Clay minerals and zeolites have been widely studied as Cs adsorbents due to their established mineralogical properties, such as the effect of surface negative charge on Cs adsorption^6–15^. The clay minerals consist of tetrahedral (T) and octahedral (O) sheets combined in a 2:1 lattice (TOT), and the isomorphic substitution in T and O sheets induces permanent negative charges through the adsorption of various cations, including Cs^13,16–18^. Zeolites as aluminosilicates consist of three-dimensional frameworks, which give them the microporous structures, in addition to the negative charges induced by isomorphic substitution in the mineral structure^7–10^. These properties of clay minerals and zeolites facilitate cationic adsorption, and various studies have investigated Cs adsorbents by focusing on the two mineral groups.

In our previous study^19^, clay minerals, such as biotite and illite, were activated by acid- and base-activations, and then used to remove Cs cation from a Cs-containing water. The acid-activation of clay minerals oxidized Fe(II) to Fe(III) in the octahedral sheet of Fe-bearing biotite, incurring mineral structure deformation, and forming frayed edge sites (FES; expanded wedge zone of clay minerals). On the other hand, base-activation of clay minerals resulted in the partial dissolution of silicon (Si) in the tetrahedral sheet, due to the high solubility of Si at alkaline pH. Moreover, the deprotonation of hydroxyl functional groups at the outer surface sites occurred, and generated pH-dependent negative charges. The experiment results of this study showed that the FES of the acid-activated biotite was more effective for Cs adsorption than the pH-dependent negative charge of base-activated illite. However, although the pH-dependent negative charge offers less Cs adsorption capacity than the FES, it is still advantageous to adsorb Cs cation.

Diatomite is a sedimentary rock mainly consisting of the fossilized diatom frustules that is made up of amorphous silica (opal-A, SiO_2_·nH_2_O)^20–24^. Besides being a low-cost material, diatomite also has unusual physico-chemical properties such as high porous structure and high mechanical strength. Hence, diatomite has widely been used as a filtration medium. Previous studies^20–23,25^ reported that diatomite was used as an adsorbent for various cations such as copper, cadmium, lead, and strontium. Other studies^24,26^ also reported that the diatomite was used to remove the radioactive Cs. Although the Cs adsorption capacity of diatomite was lower than that of other geological materials such as clay minerals and zeolite^26^, diatomite has the potential to be an effective Cs adsorbent due to the porosity and low-cost.

The Duho formation (Tertiary) in the Heunghae area of the Republic of Korea mainly consists of the siliceous mudstone containing a significant amount of diatomite (Fig. S1 of the Supplementary Information (SI))^20^. Noh et al.^20^ reported that this siliceous mudstone was composed of opal‒cristobalite/tridymite (opal‒CT) that originated from the diagenesis of diatom frustules. With the disappeared porous structure of diatom frustules by the diagenesis, this rock does not have any known commercial or industrial value. The opal-CT (SiO_2_·*n*H_2_O) originating from the diatom frustules comprises of spherical aggregates of both blade-shape cristobalite and tridymite, and hence known as lepispheres^20,27,28^. Although the highly porous structure of diatom frustules disappeared, this opal-CT also exhibits a high specific surface area (SSA)^20^ that can be made available by a chemical activation as Cs adsorbent. As mentioned above, base-activation is a more effective method for surface modification of opal-CT than acid-activation, because the opal-CT is a silica-based mineral. Base-activation will increase the SSA and improve the adsorption capacity by electrostatic attraction due to pH-dependent negative charges.

Regarding the term mudstone, in the previous studies^29–31^, mudstone was variously defined as siliceous mudstone, argillaceous mudstone, carbonaceous mudstone, etc., based on the mineralogy. Siliceous mudstone is primarily composed of silica minerals, such as quartz, opal-A, opal-CT, and feldspar; with silica content above 80%. Argillaceous mudstone mainly consists of clay minerals (e.g., illite, kaolinite), with higher content compared to silica minerals. Carbonaceous mudstone is dominated by carbonate minerals, such as calcite and dolomite. Therefore, it is necessary to clarify the definition of mudstone used in this study. The X-ray diffraction (XRD) and quantitative analyses showed that mudstones of the Duho formation were mainly composed of opal-CT (Avg.: 87.6%), with minor amounts of quartz (Avg.: 7.9%) and feldspar (Avg.: 4.5%) (Fig. S1 of the SI). Based on the previous studies, these mineral contents suggest it as siliceous mudstone^20,29–31^, however, for this study, we shall use the term opaline mudstone (OM) due to the high quantity of opal-CT, which is the primary material as a Cs adsorbent.

The objective of this study was to analyze and compare the Cs adsorption capacity of acid- and base-activated OM, and to investigate the adsorption mechanism, including the adsorption site and dynamic atomic behavior of Cs.

## Results and discussion

### Characteristics of OM

**Figure 1:**
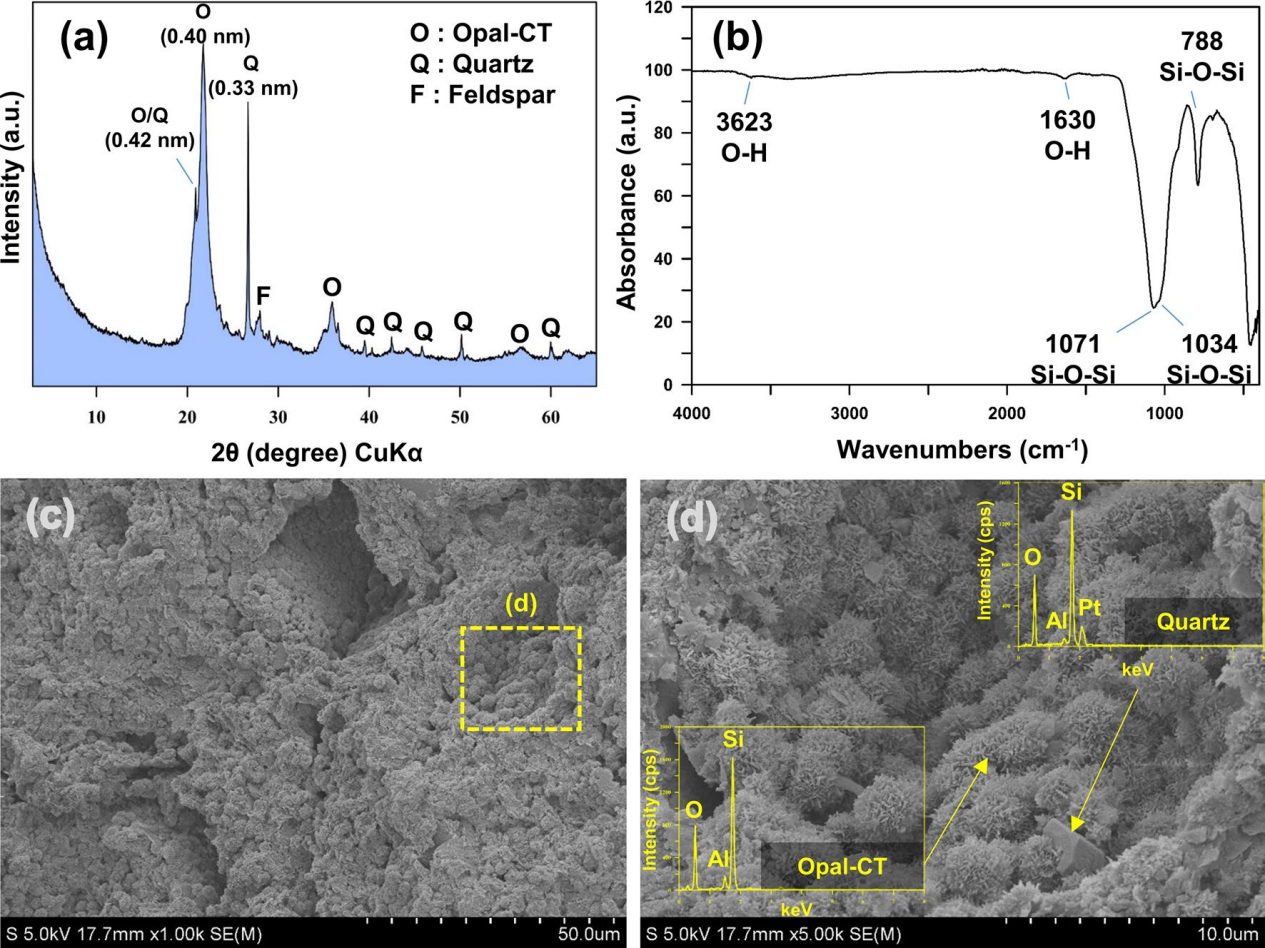
(**a**) XRD, (b) FT-IR, and (**c**,**d**) SEM–EDS analyses of the opaline mudstone.

The chemical compositions of OM as confirmed by X-ray fluorescence (XRF) were SiO_2_ (88.55%), Al_2_O_3_ (7.25%), Fe_2_O_3_ (1.51%), K_2_O (1.23%), Na_2_O (0.57%), MgO (0.45%), TiO_2_ (0.25%), and CaO (0.12%) (Total: 99.93%). The XRD results show that OM is mostly composed of opal-CT with a minor component of quartz and feldspar (Fig. 1a, and Fig. S1 of the SI). We confirmed the absorption band of OM through Fourier transform infrared spectroscopy (FT-IR) analysis at (788, 1,034, 1,071, 1,630, and 3,623) cm^−1^ (Fig. 1b). The band at 788 cm^−1^ was attributed to the Si‒O‒Si bending vibration, and intense bands at (1,034 and 1,071) cm^−1^, which represent the Si‒O‒Si stretching vibration of siloxane functional groups^23,32–34^. The bands at 1,630 and 3,623 cm^−1^ reflect the O‒H bending vibration of adsorbed water, and the stretching vibration of inner structural O‒H groups, respectively^23,32–35^. The scanning electron microscopy-energy dispersive spectroscopy (SEM–EDS) results indicated the morphology of opal-CT as lepispheres measuring about (2–3) μm each, and consisting of Si, O, and Al (Fig. 1c,d). The physico-chemical properties of OM were: SSA, 67.58 m^2^/g; cation exchange capacity (CEC), 12.21 cmol/kg; zeta potential, − 6.66 mV; mean pore diameter, 20.92 nm; total pore volume, 0.353 cm^3^/g; pH, 4.24 (Table 1).

**Table 1:** The physico-chemical properties of each activated opaline mudstone.

Activation	Non	Acid	Base
Specific surface area (m^2^/g)	67.58	101.62	107.36
Cation exchange capacity (cmol/kg)	12.21	24.44	1.09
Zeta potential (mV)	− 6.66	− 6.60	− 16.67
Mean pore diameter (nm)	20.92	14.46	14.27
Total pore volume (cm^3^/g)	0.353	0.367	0.383
pH	4.24	3.07	9.92

### Characteristics of acid- and base-activated OM

**Figure 2:**
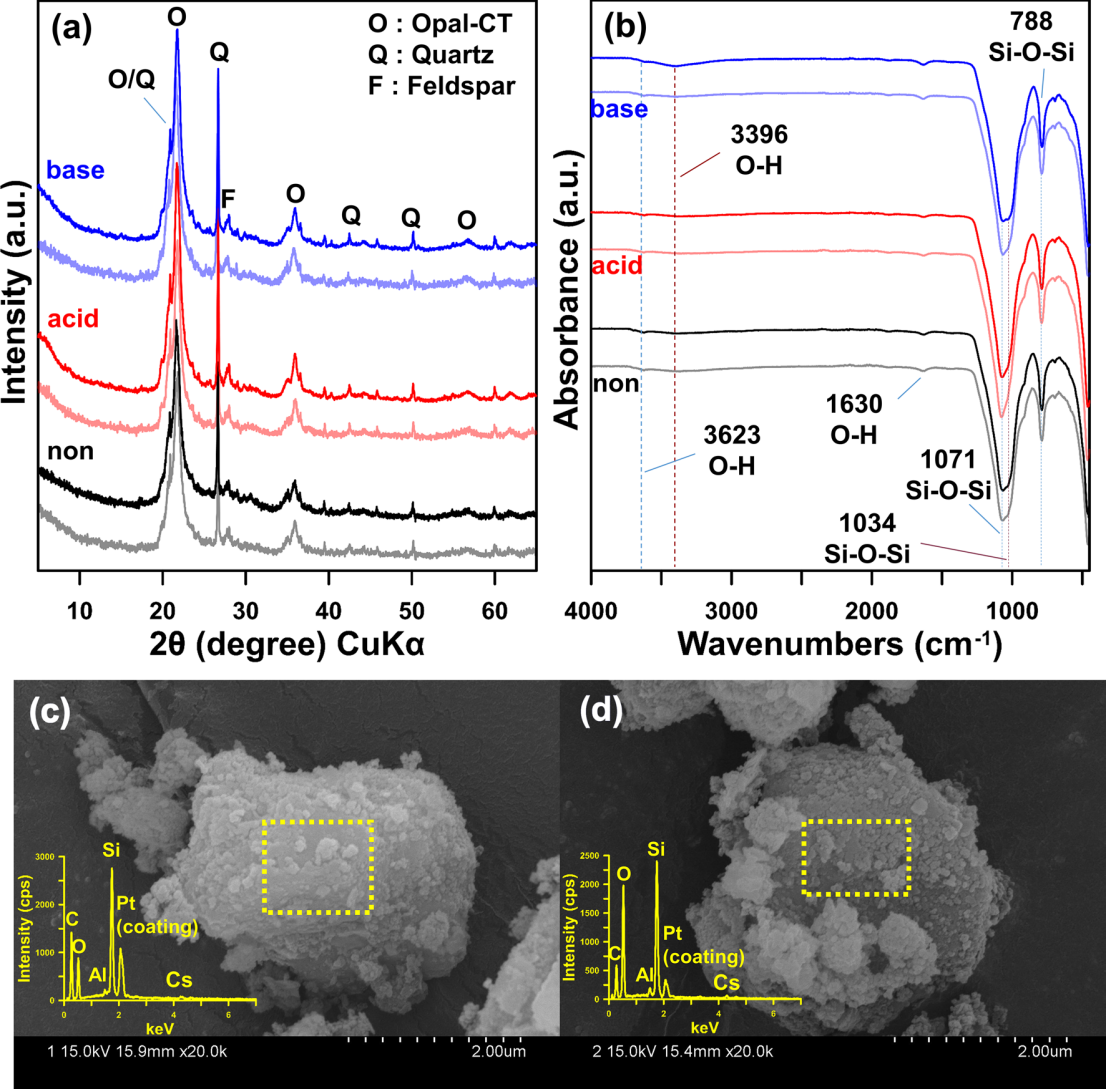
(**a**) XRD and (**b**) FT-IR analyses of all OMs, before (light) and after Cs adsorption (bold); and SEM–EDS analyses of the (**c**) acid-activated, and (**d**) base-activated, opaline mudstones after Cs adsorption.

Table 1 and Fig. 2a,b list the physico-chemical properties as determined via XRD and FT-IR of acid- and base-activated OM. The mineral phases, such as opal‒CT, quartz, and feldspar, of acid-activated OM were the same as non-activated OM (Fig. 2a), indicating that acid-activation did not affect the mineral phases of OM. In the FT-IR results, the absorption bands at 3623 cm^−1^, 3396 cm^−1^, 1630 cm^−1^, 1071 cm^−1^, and 788 cm^−1^ of acid-activated OM were similar to those of non-activated OM (Fig. 2b). However, a band at 1034 cm^−1^ represented as Si‒O‒Si stretching vibration of acid-activated OM was slightly narrow compared to that of non-activated OM. It might be that acid-activation caused the deformation of Si‒O‒Si functional groups of the mineral surface^25^. In terms of physico-chemical properties (Table 1), the SSA and CEC increased from (67.58 to 101.62) m^2^/g and (12.21 to 24.44) cmol/kg, respectively. The increased SSA and CEC results were due to the pore creation by the partial dissolution and the protonation during acid-activation, respectively^21^. The zeta potential increased slightly from (− 6.66 to − 6.60) mV, while the pH value decreased from (4.24 to 3.07). The slightly increased zeta potential was also because of the protonation^36,37^ by acid-activation. The mean pore diameter decreased from (20.92 to 14.46) nm, whereas the total pore volume increased from (0.353 to 0.367) cm^3^/g. These results indicated that acid-activation caused the partial dissolution of the mineral surface, resulting in the new pore creation, which in turn increased total pore volume^22,38^.

In the case of base-activated OM, the mineral phases such as opal‒CT, quartz, and feldspar were similar to those of non- and acid-activated OM (Fig. 2a). All absorption bands, containing a band at 1034 cm^−1^, were similar to those of non-activated OM (Fig. 2b). In terms of physico-chemical characteristics (Table 1), the SSA increased from (67.58 to 107.36) m^2^/g due to the new pore creation. The increasing rate of SSA was slightly higher than that of acid-activated OM, because the silicon (Si) solubility is high at alkaline pH^38,39^. The CEC decreased from (12.21 to 1.09) cmol/kg because of the deprotonation at the hydroxyl functional groups of the mineral surface^19^. The zeta potential decreased from (− 6.66 to − 16.67) mV, this result showed that base-activation caused the pH-dependent negative charge by the deprotonation^19,32,35^. The mean pore diameter decreased from (20.92 to 14.27) nm, whereas the total pore volume increased from (0.353 to 0.383) cm^3^/g. These results showed that new pores were created more in base-activation than in the acid-activation, and it also indicated that base-activation caused the partial dissolution of the mineral surface as in acid-activation. However, base-activation was a more effective surface modification method for increasing SSA due to high Si solubility at alkaline pH. The pH value increased from (4.24 to 9.92).

Figure 2a,b and Table 1 show that the mineral phases and IR spectra of the acid- and base-activated OMs were similar to those of the non-activated OM. However, following the chemical activation, their physico-chemical properties showed significant changes in SSA, total pore volume, and zeta potential. Summarizing the mineralogical properties as mentioned above, the chemical activation does not affect the change in the mineral phases of OM. However, the Si‒O‒Si functional groups (1034 cm^−1^) of acid-activated OM had a slightly narrower width compared with other activated OMs. According to the previous studies^21,25,32^, the siloxane and hydroxyl functional groups such as bands ranging from (1034 to 1071) cm^−1^ and (3396 to 3623) cm^−1^ were active adsorption sites. The change in these absorption bands was observed after Cs adsorption (Fig. 2b). The SSA and total pore volume of acid- and base-activated OM increased due to partial dissolution^22,38^; in particular, base-activation of OM increased the SSA and total pore volume higher than acid-activation, due to the high solubility of silica at alkaline pH^38,39^. Acid- and base-activations of OM decreased the mean pore diameter because the partial dissolution created the new pores of the mineral. The zeta potential presented a remarkable difference between acid- and base-activated OMs, showing a distinct change at a specific pH. The zeta potential of base-activated OM was significantly lower than that of non- and acid-activated OM, because of the pH-dependent negative charge at the exposed hydroxyl groups generated by the deprotonation (e.g., Si‒OH → Si‒O^−^ + H^+^)^32,36,37,40^. The CEC value of base-activated OM was lower than that of acid-activation, due to removing cations adsorbed on the mineral surface. It was also associated with the deprotonation at alkaline pH. Consequently, acid- and base-activations of OM significantly affected the physico-chemical properties of the mineral (opal-CT), but not the mineral structural characteristics.

### Cs adsorption and adsorption isotherms

**Figure 3:**
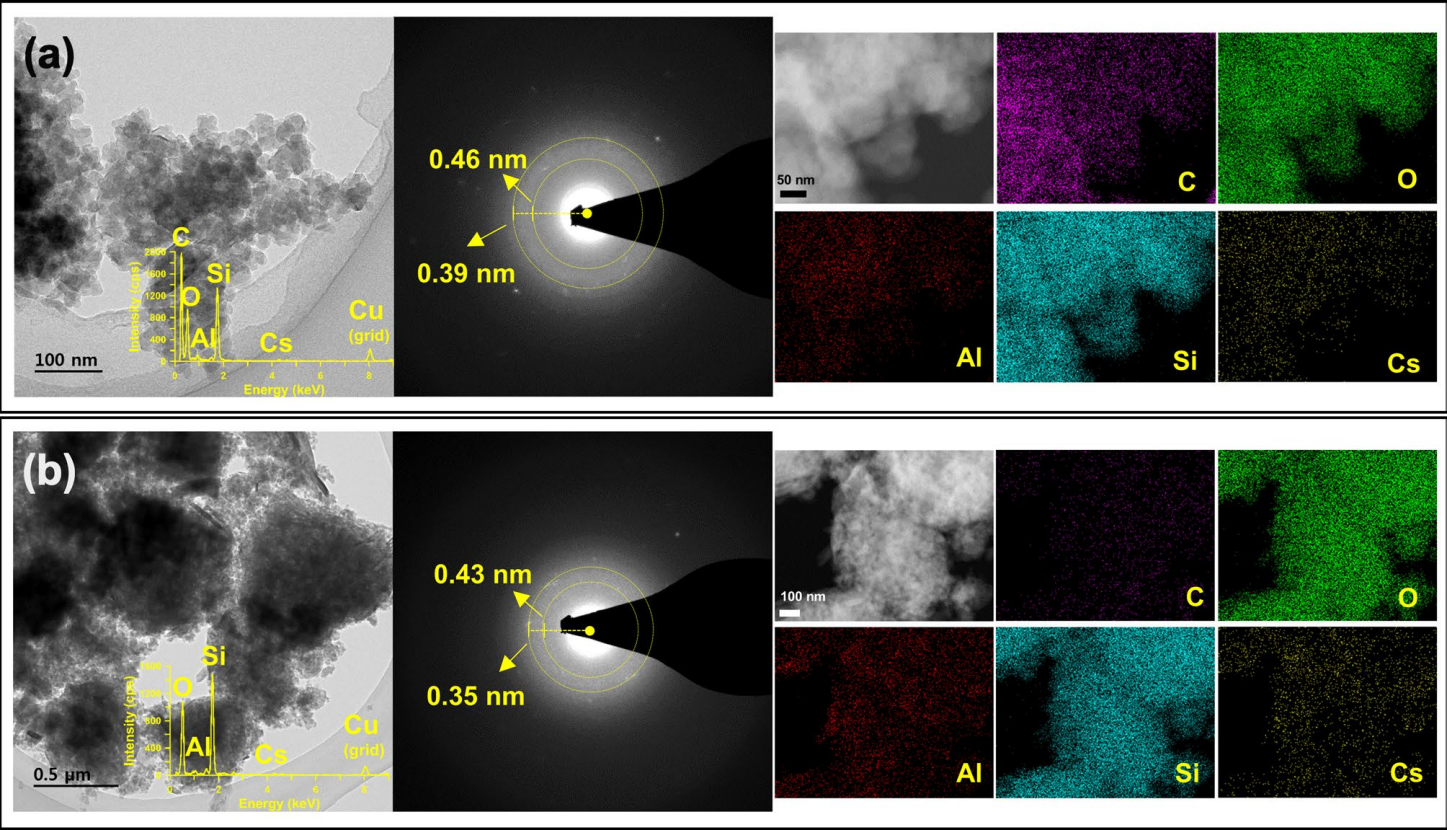
TEM-EDS images showing diffraction patterns and elemental mapping of Cs adsorption of the (**a**) acid-activated, and (**b**) base-activated, opaline mudstone.

We performed the Cs adsorption experiments on 0.1 M CsCl solution, to investigate the adsorption mechanisms, adsorption site and dynamic atomic behavior, using acid- and base-activated OM. Figure 2a shows that the mineral phases of each activated OM reacting with Cs were similar to those of all samples, indicating that the Cs adsorbed on the surface of each activated OM does not affect the mineral structure. According to the FT-IR results (Fig. 2b), the bands at 1034 and 3396 cm^−1^ of base-activated OM showed insignificant changes in width and intensity, indicating that Cs adsorption was related to the siloxane and hydroxyl functional groups. These results were similar to the adsorption sites of silicate minerals such as diatom frustules and opal reported in the previous studies^21,25,32^. We also conducted the SEM and TEM analyses to confirm the Cs adsorbed on the opal-CT. We conducted the SEM–EDS analyses to confirm the adsorption of Cs cation on the surface of opal-CT in acid- and base-activated OMs (Fig. 2c,d). In addition to SEM–EDS, the diffraction pattern and elemental mapping results in TEM–EDS were used to validate the Cs adsorption on the surface of opal-CT (Fig. 3). The results of TEM–EDS showed spherical aggregates of opal-CT composed of Si, O, and Al in each activated OM, and confirmed the adsorption of Cs with 0.8 wt. % (acid) and 2.8 wt. % (base). The base-activated OM showed higher Cs adsorption capacity than acid-activated OM. In addition, the diffuse diffraction patterns indicated an amorphous mineral^41^, i.e., opal-CT, and its *d*-spacing ranged (0.35 to 0.46) nm. These values were similar to the XRD results (Fig. 1a). The elemental mapping images also showed the adsorption of Cs cations on the surface of opal-CT. Therefore, the results of FT-IR, SEM, and TEM suggested that Cs cation adsorbed to the surface of opal-CT at the siloxane and hydroxyl functional groups.

**Figure 4:**
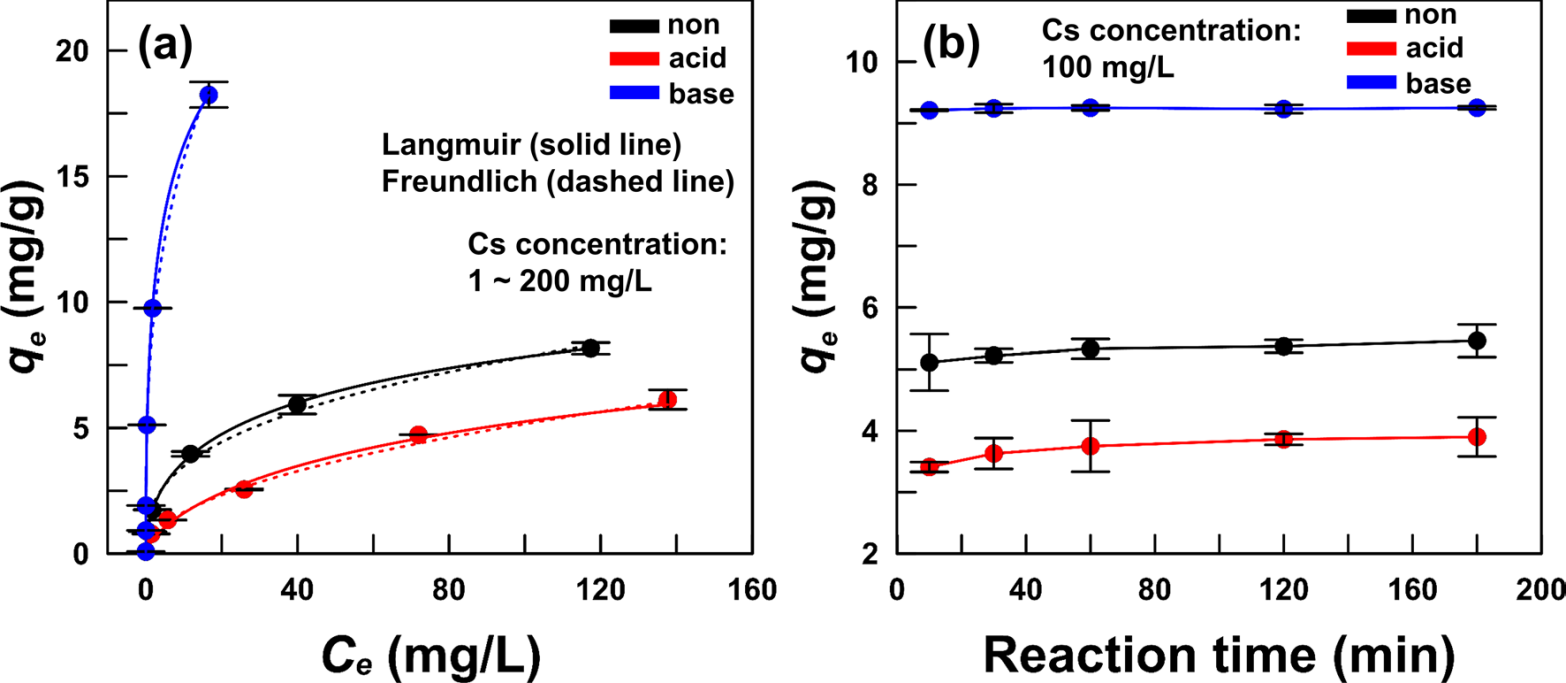
(**a**) Adsorption isotherms, and (**b**) the effect of Cs adsorption reaction time on each activated opaline mudstone.

Table 2 and Fig. 4a show the adsorption isotherm and its parameters in the Langmuir and Freundlich models. The correlation coefficients (*R*^2^) based on the adsorption isotherms of all activated OMs were approximately 0.99. Based on the Langmuir model, the acid-activation of OM slightly decreased the adsorption capacity (*q*_*m*_) from (15.47 to 12.22) mg/g, while the base-activation of OM largely increased the *q*_*m*_ value from (15.47 to 32.14) mg/g. These results indicate that base-activation further enhanced the Cs adsorption capacity of OM more as compared to acid-activation, and it corresponded to the TEM–EDS results (acid: 0.8 wt.%; base: 2.8 wt.%). Based on the Freundlich model, the acid-activation of OM decreased *K*_*F*_ from (1.55 to 0.53) (mg/g)/(mg/L)^1/n^, while the base-activation of OM largely increased the value from (1.55 to 7.39) (mg/g)/(mg/L)^1/n^. These results demonstrated that the Cs adsorption of base-activated OM fitted with both the Langmuir (monolayer) and Freundlich (multilayer adsorption) models, making it base-activated OM a high potential candidate as an effective Cs adsorbent.

**Table 2:** Langmuir and Freundlich model parameters for Cs adsorption on each activated opaline mudstone.

Activation	Langmuir			Freundlich		
	*K*_*L*_ (L/mg)	*q*_*m*_ (mg/g)	*R*^2^	*K*_*F*_ (mg/g)/(mg/L)^1/n^	*n*	*R*^2^
Non	0.09	15.47	0.99	1.55	2.84	0.99
Acid	0.03	12.22	0.98	0.53	2.02	0.99
Base	0.33	32.14	0.99	7.39	3.06	0.99

Figure 4b presents the effect of reaction time on each activated OM. Among the three OMs, the base-activated OM showed the highest Cs adsorption capacity of about 9.23 mg/g (removal efficiency: 97.9%). In addition, the Cs adsorption also rapidly reached equilibrium within 10 min. Unlike the base-activated OM, the non- and acid-activated OM showed low Cs adsorption capacity of about (5.29 and 3.71) mg/g with removal efficiency of (56.1 and 39.5) %, respectively. The Cs adsorptions for the non- and acid-activated OM reached equilibrium after 2 h, suggesting a lower adsorption rate than the base-activated OM.

To confirm the adsorption performance of base-activated OM, we compared the Cs adsorption capacity of base-activated OM with the other geological material (e.g., clay minerals, zeolite, and other minerals, etc.) based adsorbents (Table 3). The Cs adsorption capacity of base-activated OM was 32.14 mg/g, which is higher than 6.02 mg/g of acid-activated biotite and 13.21 mg/g of base-activated illite, but lower than 35.77 mg/g of Mg-vermiculite, as presented in our previous study^19^. Although the Cs adsorption capacity of base-activated OM was slightly lower than that of Mg-vermiculite, the base-activated OM is an eco-friendly geological material produced by a simple surface modification method.

**Table 3:** Comparison of Cs adsorption capacity with the different geological mineral adsorbents.

Adsorbent	Adsorption capacity (mg/g)	References
Acid-activated opaline mudstone	12.22	This study
Base-activated opaline mudstone	32.14	
Acid-activated biotite	6.02	Kwon et al.^19^
Base-activated illite	13.21	
Chabazite	1.86	Borai et al.^7^
Sericite	6.68	Kim et al.^50^
Activated sericite	4.35	Tiwari et al.^11^
Zeolite Rho	3.47	Lee et al.^10^
NH_4_-vermiculite	4.11	Yin et al.^12^
K-vermiculite	9.16	
Mg-vermiculite	35.77	
Chitosan-grafted magnetic bentonite	1.21	Yang et al.^66^

**Figure 5:**
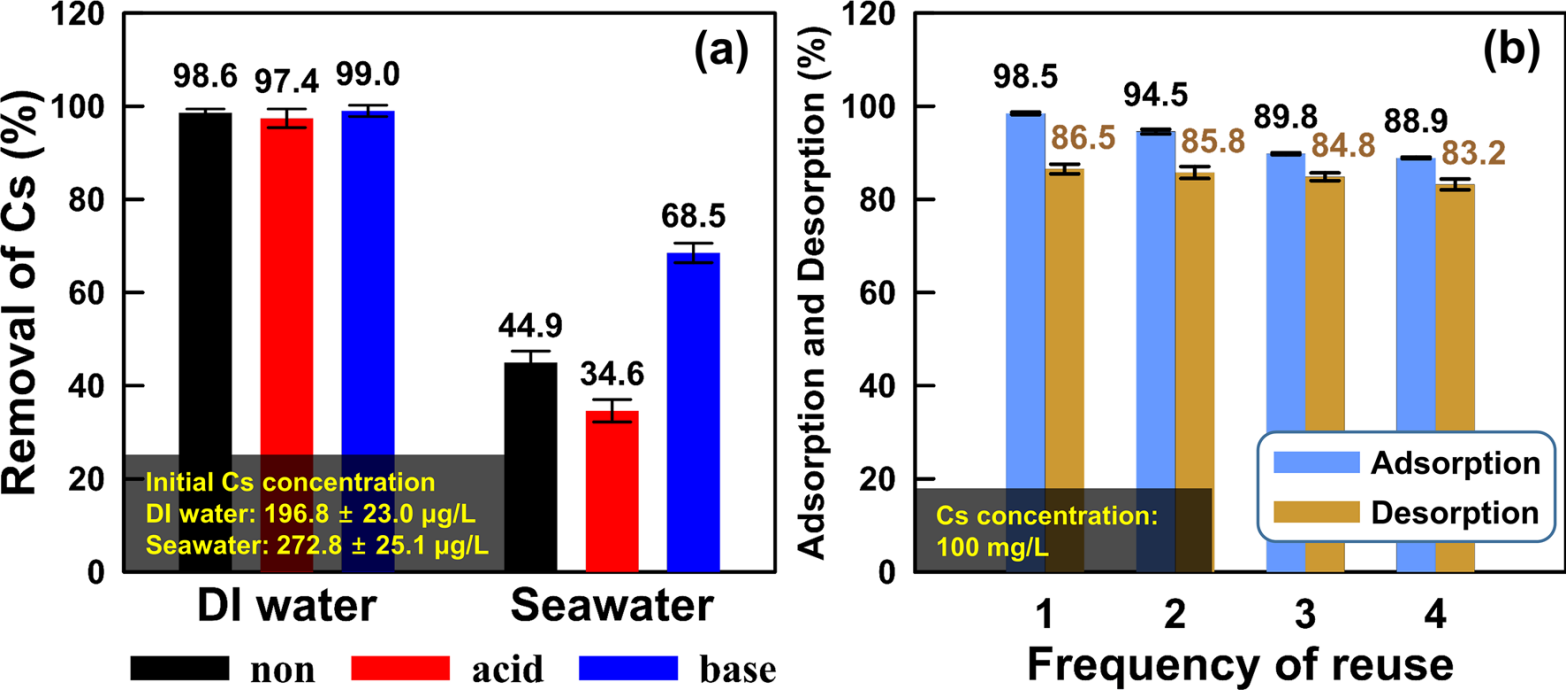
The Cs removal efficiency of Cs-containing DI water and seawater using (**a**) each activated opaline mudstone, and (**b**) the frequency of reuse using base-activated opaline mudstone.

To simulate the natural environmental conditions, we performed the Cs adsorption experiments using low concentrations of Cs-containing DI water (average initial concentration: (196.8 ± 23.0) μg/L) and seawater (average initial concentration: (272.8 ± 25.1) μg/L) (Fig. 5a). In the case of Cs-containing DI water, the Cs removal efficiencies and the *K*_*d*_ values of each activated OM for non, acid, and base were (98.6, 97.4, and 99.0) %, and (4,421.1, 2,419.7, and 6,771.8) mL/g, respectively. Previous studies suggested that a *K*_*d*_ value higher than 5,000 indicated an excellent and appropriate adsorbent^42,43^. Although the Cs removal efficiencies of the activated OMs were almost equal, the *K*_*d*_ values showed that base-activated OM had higher Cs adsorption capacity than the others. However, the *K*_*d*_ value of adsorbents used for Cs removal from the Cs-containing DI water was practically insignificant because only one Cs cation existed in DI water. Therefore, we conducted an experiment to confirm the *K*_*d*_ value and Cs removal efficiency of each activated OM from seawater containing various high concentrated cations, specifically, Na (8,515.1 mg/L), Mg (1,011.6 mg/L), K (315.7 mg/L), and Ca (312.1 mg/L). In the Cs containing seawater, the Cs removal efficiencies of all activated OMs for non, acid, and base decreased to (44.9, 34.6, and 68.5) %, respectively, owing to the high concentration of competing cations, such as Na, Mg, K, and Ca^44,45^. The Cs cation *K*_*d*_ value of each activated OM for non, acid, and base also decreased to (81.8, 53.1, and 218.5) mL/g, respectively. Among these activated OMs, only the base-activated OM had a high *K*_*d*_ value of 218.5 mL/g, higher than those of the highly concentrated cations of Na (0.60 mL/g), Mg (0.45 mL/g), K (3.28 mL/g), and Ca (0.16 mL/g). These results are believed to be due to two factors; relative charge density (Eq. charge density = total charge / total volume)^17^ and hydrated ionic radius. The relative charge density of Cs cation (1.00 e/nm^3^, based on the charge density of hydrated Cs cation) was higher than those of other cations (Na: 0.78 e/nm^3^, Mg: 0.91 e/nm^3^, K: 0.98 e/nm^3^) except for Ca of 1.02 e/nm^3^. However, although the Ca cation has a higher relative charge density than the Cs cation, the *K*_*d*_ value of Ca cation was lower than that of Cs cation. This result was considered that the difference in hydrated ionic radius. The Cs cation has the lowest hydrated ion radius (0.329 nm)^13,46^ compared with other cations (Na: 0.358 nm, Mg: 0.428 nm, K: 0.331 nm, Ca: 0.412 nm). Therefore, higher relative charge density and lower hydrated ionic radius of Cs cation, compared to other high concentrated cations, are regarded as advantageous for adsorption onto the mineral surface of base-activated OM.

Regarding the main Cs adsorption mechanism, the SSA, mean pore diameter, and total pore volume of OM were almost equal for both acid- and base-activation (Table 1). In addition, in the case of CEC, acid-activated OM was higher than base-activated OM. However, the zeta potential of the base-activated OM was − 16.67 mV, which was much lower than the − 6.60 mV of the acid-activated OM (Table 1). This means that the pH-dependent negative charge generated via the deprotonation under alkaline pH is the main mechanism for Cs adsorption. The maximum Cs adsorption capacity of the base-activated OM was also higher compared with the other geological mineral adsorbents reported in the previous studies (Table 3).

### Cs desorption and reusability

The Cs desorption from the Cs adsorbent is as essential as the Cs adsorption behavior by the Cs adsorbent. The previous studies^47–50^ reported that the Cs adsorbents have to be reusable to reduce cost and maximize utilization. Therefore, to evaluate the potential for reusability of base-activated OM, we conducted the Cs desorption experiments. The Cs desorption efficiency of the base-activated OM reacting with Cs cation was confirmed using 0.1 M HCl and 0.1 M NaOH solutions, i.e., acid and base activations, as 87.2%, and 77.2%, respectively (Fig. S2 of the SI). Base-activation was expected to increase the Si solubility and Cs desorption efficiency, but the acid-activation was more effective for Cs desorption than the base-activation. These results could be explained by the higher relative charge density (based on the charge density of hydrated Cs cation) of H_3_O^+^ (1.62 e/nm^3^) in acid solution, than that of Cs cation (1.00 e/nm^3^); making it more advantageous in competition for adsorption sites. Therefore, acid-activation has to be used to desorb the Cs cations from the mineral surface of the base-activated OM.

Figure 5b shows the Cs adsorption and desorption efficiencies of the reused base-activated OM. The Cs adsorption efficiency decreased with the frequency of reuse (first, second, third, and fourth) as (98.5, 94.5, 89.0, and 88.9) %, respectively, and the reduced rate up to three uses showed an average value of 4.7%. The Cs desorption efficiency was also decreased with the frequency of reuse (first, second, third, and fourth) as (86.5, 85.8, 84.8, and 83.2) %, respectively; with an average reduction rate of 1.1%. These results indicate that base-activated OM has high Cs adsorption capacity and reusability.

### Cs adsorption mechanisms

The previous studies^21,22,32,33,38^ explained that the high Cs adsorption capacity of diatomite was due to the high SSA by the mesoporous property and the surface negative charge caused at the alkaline pH. This study also showed that the OM has a high SSA of 67.58 m^2^/g and a negative zeta potential of − 6.66 mV (Table 1). However, the Cs removal efficiency of OM from the Cs-containing seawater was 44.9% (Fig. 5a), low and ineffective. To enhance this, we used acid- and base-activations. First, the acid-activation of OM highly increased the SSA (101.62 m^2^/g), due to the new pore creation (increased total pore volume) by acid-activation, and the zeta potential of acid-activated OM was − 6.60, which is similar to non-activated OM (Table 1). The acid-activated OM was expected to increase the Cs removal efficiency from the Cs-containing seawater; however, it had a lower Cs removal efficiency than non-activated OM (Fig. 5a). This result indicates that increase in SSA cannot guarantee improvement of the Cs removal efficiency, and the formation of adsorption sites cannot explain the Cs adsorption mechanism. On the other hand, base-activated OM showed higher the Cs removal efficiency (68.5%) from the Cs-containing seawater than non- and acid-activated OM (Fig. 5a). With respect to the physico-chemical properties of acid- and base-activated OM, the change in the zeta potential showed significant difference (Table 1). The zeta potential of base-activated OM was − 16.67 mV, indicating that the mineral surface of base-activated OM was negatively charged more than non- and acid-activated OM, because of the deprotonation by base-activation. Thus, the pH-dependent negative charge, rather than the high SSA, of base-activated OM was the primary Cs adsorption mechanism.

**Figure 6:**
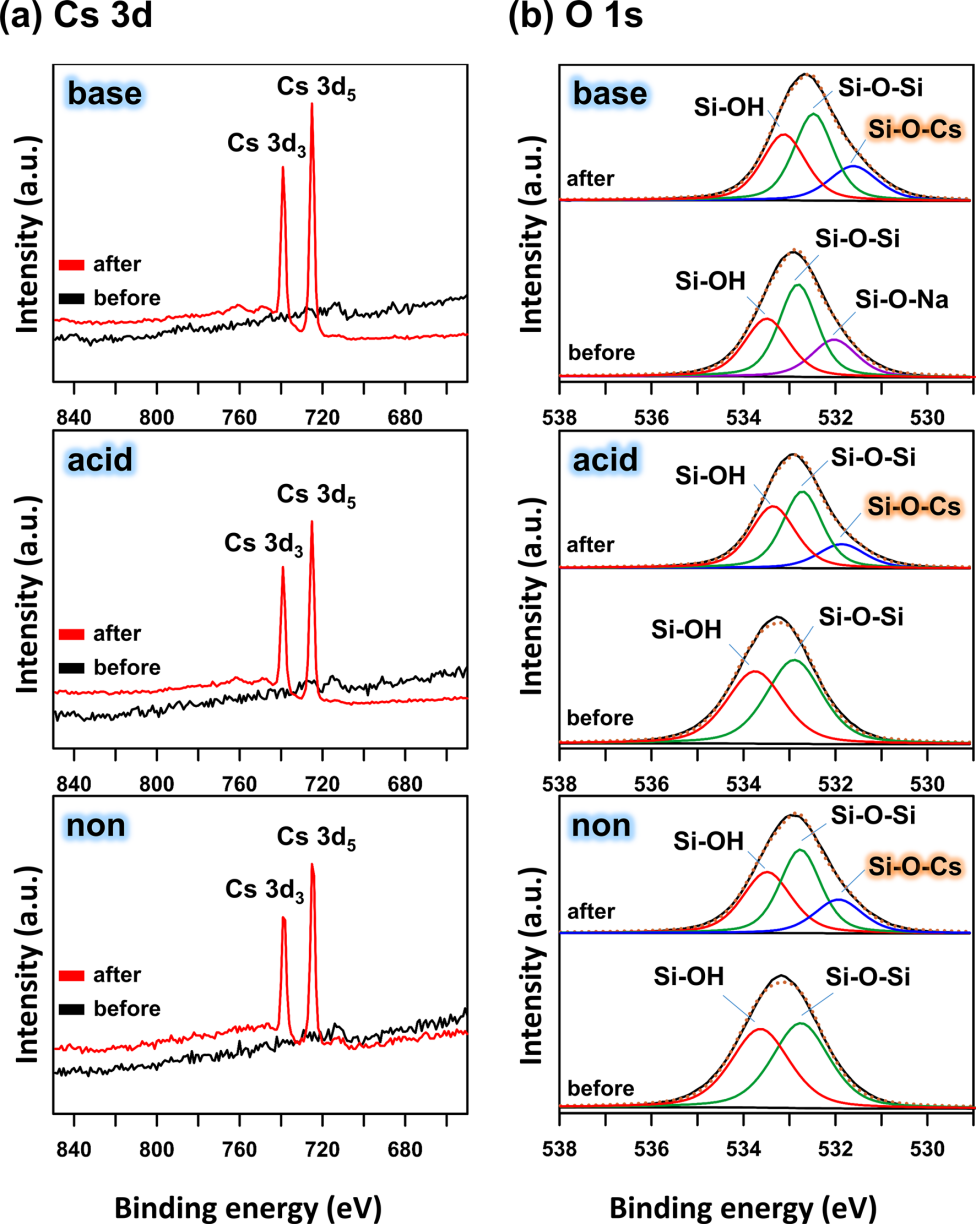
The XPS spectra of (**a**) Cs 3d and (**b**) fitted O 1 s (background subtracted), of each activated opaline mudstone before and after Cs adsorption.

The siloxane and hydroxyl functional groups, as the adsorption sites, were explained by the previous studies^21,32,33^. In the FT-IR results (Fig. 2b), among the absorption bands of base-activated OM, the bands at 3396 cm^−1^ and 1034 cm^−1^ showed the change after Cs adsorption. Therefore, based on the previous studies^21,32,33^ and our analysis results, the Cs cation adsorbed on the mineral surface may exist on the Si‒O‒Cs forms. Various studies reported that the Si‒OH, Si‒O‒Si, and Si‒O‒Cs bonds were confirmed via deconvolution of the O 1 s spectrum of the X-ray photoelectron spectroscopy (XPS) results^51,52^. Therefore, to investigate the chemical bonding such as Si‒O‒Cs and Cs adsorption, we performed the XPS analysis. The binding energies of (725.0 and 738.9) eV base- and acid- activated OM after the Cs adsorption corresponded to Cs 3d_5_ and Cs 3d_3_, respectively (Fig. 6a). The O 1s spectrum of each activated OM and the same sample reacting with the CsCl solution were deconvoluted into two or three peaks, Si‒OH, Si‒O‒Si, or Si‒O‒Cs, in order to elucidate the chemical bonding. The binding energy of Si‒OH was higher than that of Si‒O‒Si^52^, due to the difference in Pauling electronegativity values (H: 2.20, Si: 1.90), while the Na and Cs exhibited low Pauling electronegativity (Na: 0.93, Cs: 0.79), and thus the binding energies of Si‒O‒Na (formed by base-activation) and Si‒O‒Cs were lower than that of Si‒O‒Si^53,54^. Accordingly, their binding energies were high in the order Si‒OH, Si‒O‒Si, Si‒O‒Na or Cs, corresponding to (533, 532, and 531) eV, respectively^51,52^. The deconvolution results of O 1s spectra of each activated OM were as shown in Fig. 6b. The binding energies and quantities of Si‒OH and Si‒O‒Si of non-activated OM were (533.6 and 532.8) eV at (48.6 and 51.4) %, respectively. For the acid-activated OM, the binding energy of Si‒OH and Si‒O‒Si increased slightly to (533.8 and 532.9) eV, respectively, but the quantity of Si‒OH was lower than that of non-activated OM, while that of Si‒O‒Si was higher than that of the non-activated OM. Compared with the non- and acid-activated OM, the O 1s of base-activated OM can be deconvoluted into three peaks; Si‒OH, Si‒O‒Si, and Si‒O‒Na. The Si‒O‒Na peak was attributed to the reaction between Si‒O‒Si and NaOH during the base-activation (e.g., Si‒O‒Si + NaOH → Si‒OH + Si‒O‒Na)^51,55^. The Si‒OH and Si‒O‒Si peaks of the base-activated OM showed similar binding energy to that of the non-activated OM, and Si‒O‒Na showed a lower binding energy of 532.0 eV (21.7%), compared to the others. In the case of Cs-adsorbed OM, the binding energies of all functional groups decreased compared to the non-reacted OM, and Cs cation was adsorbed as the Si‒O‒Cs. The Si‒O‒Cs levels of the Cs-adsorbed sample were 16.6% (acid) and 21.2% (base). The difference in Si‒O‒Cs content of the Cs-adsorbed sample showed that the base-activated OM carried a higher Cs adsorption capacity than the acid-activated OM. These results were consistent with those of adsorption experiment.

Based on the results of FT-IR, SEM, TEM, and XPS, the Cs cation was adsorbed on the Si–O functional groups of opal-CT (Figs. 2, 3, and 6). However, the previous study^23^ explained that the absorption band ranging from 3000 to 4000 cm^−1^ of the FT-IR is attributed to the physically adsorbed water molecules. In contrast, other studies^32,33^ explained that the band at 1630 cm^−1^ represents the bending vibration of adsorbed water molecules. Therefore, the Cs atomic dynamical behavior, of the water molecules, of the Cs adsorption by base-activated OM is necessary for the clarity of Cs adsorption mechanisms. Kim et al.^17^ confirmed the dynamic behavior, such as outer-sphere complexes (OS) and inner-sphere complexes (IS) of the adsorbed Cs, via ^133^Cs MAS NMR analysis. The IS-type Cs indicated that Cs cation is directly and tightly bound to the mineral surface (Stern layer), without water molecules. On the other hand, the OS-type Cs cation is loosely bound to the mineral surface (diffuse layer) with water molecules. The Cs dynamic behavior is highly affected by the amount of water on the surface (e.g., room humidity)^17^. Increase in humidity leads to overlap of the IS and OS-type Cs peaks in NMR analysis into a single indistinguishable intense peak. Therefore, the wet and dry base-activated OMs reacting with CsCl solution were prepared in the range of room temperature (RT) to 110 °C for 1 h, respectively.

**Figure 7:**
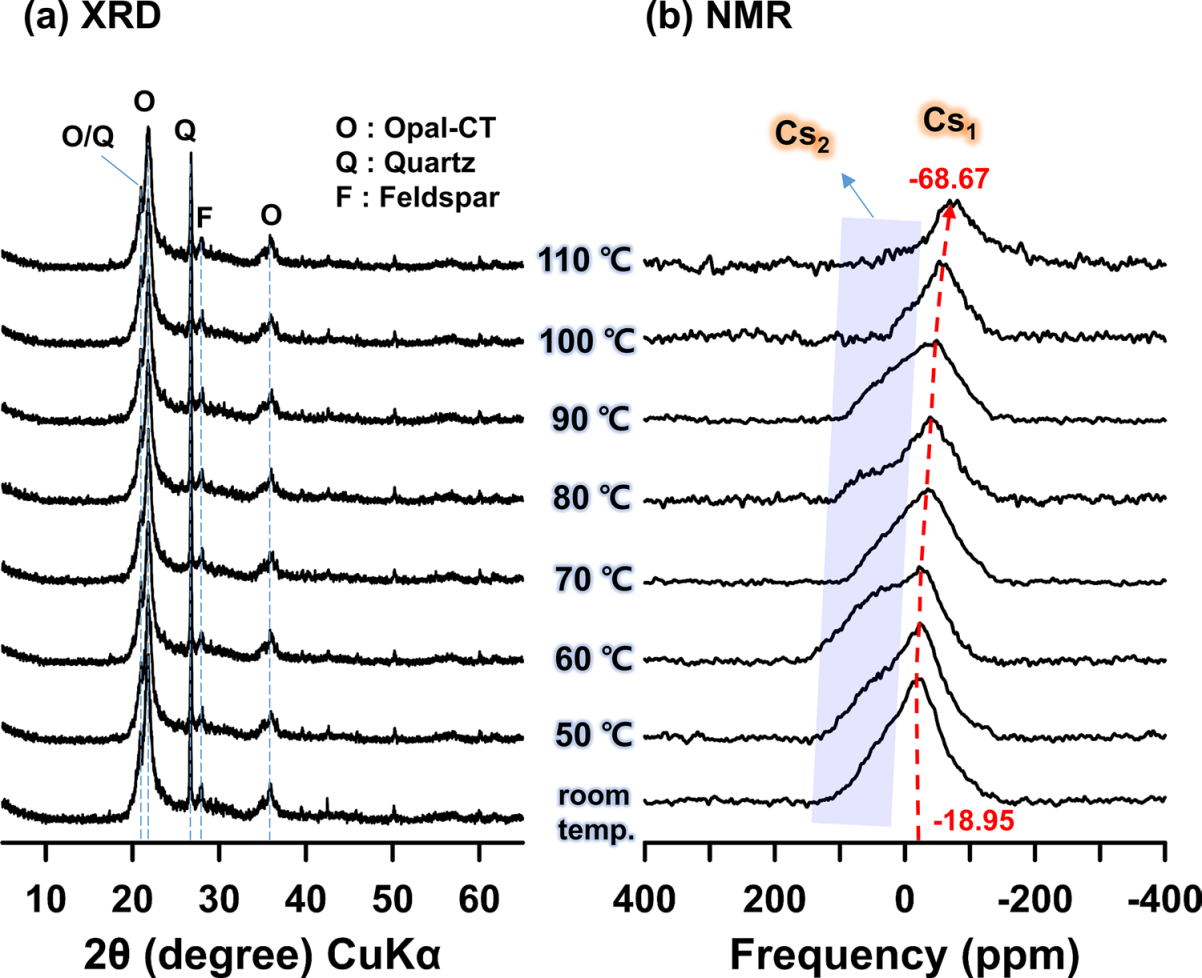
The (**a**) XRD, and (**b**) ^133^Cs MAS NMR spectra of base-activated opaline mudstone, after Cs adsorption at temperatures from RT to 110 °C.

**Figure 8:**
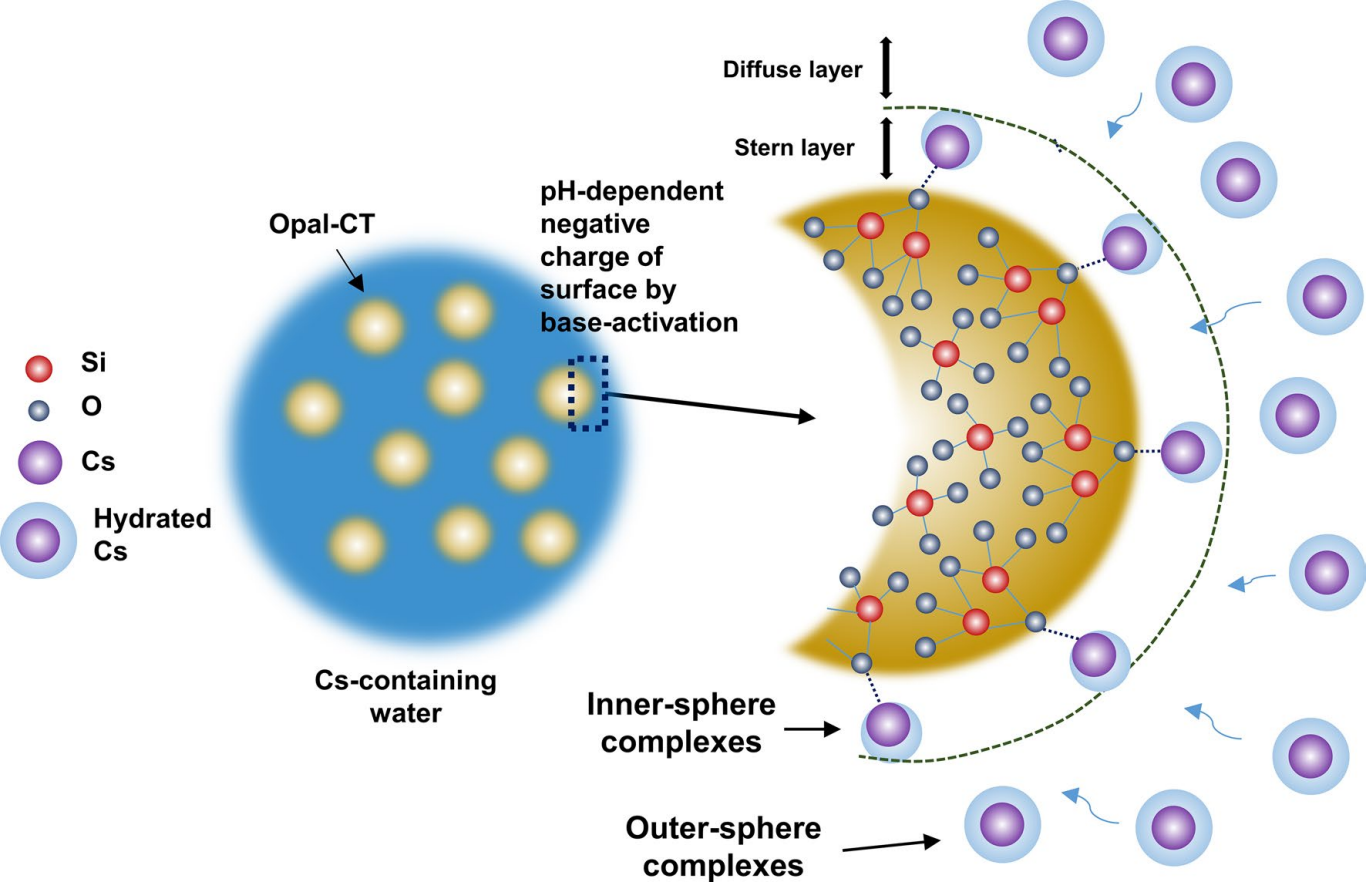
Schematic of the Cs adsorption mechanisms and dynamic behavior of the base-activated opaline mudstone (Si − O functional groups and hydrated Cs images modified from Khraisheh et al.^32^ and Lee et al.^13^.

The XRD results showed that the mineral phase and intensity of the heated OMs were similar to those of the non-heated OM (Fig. 7a), indicating no change in the mineral structure by increased temperature. In the NMR results (Fig. 7b), the peak of non-heated OM distinctly appeared at − 18.95 ppm, and had a wide width, indicating that Cs cation was adsorbed on the mineral surface in at least two environments (i.e., IS and OS)^17^. Upon heating, the peak position for Cs_1_ gradually decreased (was more shielded) from (− 18.95 to − 68.67) ppm, and the peak intensity also decreased, compared with those at RT. The Cs_2_ (Fig. 7, blue rectangle area) showed a large change in peak position and intensity. In particular, at temperatures from (100 to 110) °C, the broad peaks disappeared, indicating that Cs_1_ was directly and tightly bound to the basal oxygen (IS-type Cs), and Cs_2_ was loosely bound to the basal oxygen with water molecules (OS-type Cs). However, according to Kim et al.^17^, the peak of OS-type Cs was distinctly separate from the IS-type Cs peak. Since Cs cation had low hydration energy^13^, when Cs cation was directly adsorbed to the basal oxygen, water molecules could partially exist in Cs cation, except for the part bound to basal oxygen. In other words, the adsorbed Cs cation mainly existed as IS-type, not OS-type. NMR and XPS analyses suggest that the hydrated Cs cation in the Cs-containing water was directly and tightly bonded to the oxygen in the Si‒O functional groups (Stern layer), and was partially bonded with water molecules (Fig. 8).

## Materials and methods

### Preparation of materials and chemicals used activation

The OM was collected from the Duho formation (Tertiary) of the Heunghae area in Pohang city, Republic of Korea (Fig. S1 of the SI). The collected sample was pulverized using a bowl mill grinder to a particle size of less than 75 μm with a sieve (200 mesh). We then analyzed the characteristics of the powdered sample for application in Cs adsorption.

The acid and base activations were performed using hydrochloric acid (HCl, 35%, OCI) and sodium hydroxide (NaOH, 98.0%, Daejung), respectively. The Cs adsorption experiments were carried out using cesium chloride (CsCl, 99.0%, Samchun), which has similar chemical characteristics to radioactive ^137^Cs^13,56^. The CsCl stock solution was obtained by mixing 1.2667 g of CsCl with 1 L of distilled (DI) water (1,000 mg/L of Cs concentration), and diluting with both DI water and seawater to reduce the concentration of Cs. We then used Cs-containing seawater and DI water to analyze the Cs adsorption capacity and the removal efficiency of each activated sample. The seawater was obtained from the coastal sea area of Yeosu in Republic of Korea.

### Characterization of OM

The chemical composition of OM was analyzed via X-ray fluorescence (XRF, Axios Minerals, PANalytical) at 50 kV and 40 mA. The mineral phases were confirmed using X-ray diffraction (XRD, Empyrean, PANalytical) with the the CuKα radiation at 40 kV, 30 mA, and 0.01° of 2θ step. The morphology and chemistry were determined by scanning electron microscopy (SEM, SU-70, Hitachi) with energy dispersive electroscopy (EDS) at an accelerating voltage ranging (5–20) kV. The chemical bonding was determined using Fourier transform infrared spectroscopy (FT-IR, Spectrum 400, PerkinElmer). The chemical bonding and Cs adsorption were analyzed via X-ray photoelectron spectroscopy (XPS, K-Alpha + , Thermo Scientific) with the AlKα radiation (1486.6 eV) at a high-resolution of 0.1 eV step, and the binding energy was calibrated to 284.6 eV (C 1s). The O 1 s peaks were fitted at a Gaussian–Lorentz peak shape (G:L ratio = 60:40) and Shirley background function. The SSA, pore diameter, and pore volume were determined via the Brunauer–Emmett–Teller method (BET, BELSORP mini II, BEL Japan) with N_2_ adsorption–desorption isotherms at 77 K. The cation exchange capacity (CEC) was evaluated with 1 M ammonium acetate at pH 7^57^. The zeta potential was analyzed using the electrophoretic light scattering method with ELS-800 (Otsuka) at RT.

### Characterization of acid- and base-activated OM

The characterization of acid- and base-activated OM was performed using the same analyzer and method for non-activated OM. However, different analyzers were used to characterize each activated OM and after Cs adsorption. Transmission electron microscopy (TEM, Tecnai F20, FEI) with diffraction pattern and elemental mapping was used to determine the mineralogy, chemistry, and Cs adsorption state of each activated OM reacting with the CsCl solution, at an accelerating voltage of 200 kV. The dynamic atomic behavior of Cs on the mineral surface, including the outer-sphere complexes (OS) and inner-sphere complexes (IS), was analyzed via 400 MHz solid-state ^133^Cs MAS NMR (AVANCE III HD, Bruker). The NMR spectrum fitting was performed with a Lorentz peak shape and linear background function.

### Acid- and base-activations

The acid- and base-activations with HCl and NaOH utilized in this study are similar to those for chemical activation with HCl and NaOH, as previously reported in our study^19^. Note that the mineralogical properties, such as the SSA, surface charge, CEC, and total pore volume, and the Cs adsorption capacity were different for the acid- and base-activations. In particular, base-activation of silica-based material increased the Si solubility, and deprotonation of exposed hydroxyl functional groups, generating the pH-dependent negative charge. Based on these results, when the OM composed of opal-CT was activated using the alkaline solution, we expected it to increase the SSA, and generate pH-dependent negative charge. In addition, we also conducted acid-activation and compared it with base-activation, to determine the most effective method for the Cs adsorption capacity of OM. The chemical activation methods based on the study of Kwon et al.^19^ were as follows: the acid-activation was conducted by mixing 10 g of powdered sample with 100 mL of 0.1 M HCl, and then stirring the mixture for 10 min at RT, followed by separation of the activated sample from the solution through centrifugation at 6000 rpm for 15 min. The separated sample was washed with DI water three times to remove the residual acid, and then oven-dried at 60 °C. The base activation was also carried out using the same procedure as the acid activation but with 0.1 M NaOH. Each dried activated sample was ground, before conducting the experiments. Figure S3a of the SI shows the procedures for acid and base activations.

### Cs adsorption and adsorption isotherms

The previous studies^4,5^ reported that the ^137^Cs concentration of radioactive waste water from Fukushima in Japan was less than 1 mg/L. Hence, various studies investigating Cs adsorption were conducted at a Cs low concentration (e.g., (0.2–0.25), and 1 mg/L)^4,19,42,45,58^. In this study, we performed the Cs adsorption experiments using approximately 0.25 mg/L Cs-containing DI water or seawater to simulate Cs removal in the natural environment. In the case of other experiments, such as adsorption isotherms and sites, and dynamic atomic behavior, the Cs concentration was higher, compared to the adsorption experiments under simulated natural environments.

For the Cs adsorption experiments, 0.2 g of sample was mixed with 20 mL of Cs-containing water in a 50 mL conical tube. The mixture was then agitated using a shaker at 150 rpm for 2 h at RT. The supernatant was separated from the mixture via centrifugation at 6,000 rpm for 15 min, and then filtered using a 0.2 μm syringe filter. The residual Cs concentration in the supernatant was analyzed via inductively coupled plasma mass spectrometry (ICP-MS, NexION 300, PerkinElmer). All Cs adsorption experiments in this study were performed in triplicates using average values of the initial and residual Cs concentrations. The Cs adsorption capacity (*q*_*e*_, mg/g), removal efficiency (%), and distribution coefficients (*K*_*d*_, mL/g) were calculated as follows:

1 $$ {\text{Adsorption}}\;{\text{capacity}}\;\left( {\text{mg/g}} \right) \, = \frac{{\left( {C_{0} - C_{e} } \right)V}}{M} $$

2 $$ {\text{Removal}}\;{\text{efficiency}}\;\left( \% \right)\frac{{C_{0} - C_{e} }}{{C_{0} }} \times 100 $$

3 $$ {\text{Distribution}}\;{\text{coefficients}}\;\left( {\text{mL/g}} \right) \, = \frac{{\left( {C_{0} - C_{e} } \right)}}{{C_{e} }} \times \frac{V}{M} $$

where, *C*_*0*_ (mg/L) and *C*_*e*_ (mg/L) denote the initial and equilibrium concentrations of Cs, respectively; *V* (L) is the volume of solution, and *M* (g) is the weight of adsorbent. Figure S3b of the SI shows the mechanisms of Cs adsorption using each activated sample.

Langmuir and Freundlich adsorption isotherms were used to confirm the Cs adsorption behavior. The Langmuir adsorption isotherm represents monolayer adsorption, where a single molecule is adsorbed onto a single adsorption site, while the Freundlich adsorption isotherm highlights multilayer adsorption, involving several adsorption sites on the adsorbent surface^42,44,59–65^. The nonlinear and linear equations associated with these adsorption isotherms are calculated as follows:

4 $$ {\text{Langmuir}}:\;q_{e} = q_{m} \frac{{K_{L} C_{e} }}{{1 + K_{L} C_{e} }} $$

5 $$ {\text{Langmuir}}\;\left( {{\text{linear}}} \right):\frac{1}{{q_{e} }} = \frac{1}{{q_{m} K_{L} }}\frac{1}{{C_{e} }} + \frac{1}{{q_{m} }} $$

6 $$ {\text{Freundlich}}:\;q_{e} = K_{F} C_{e}^{1/n} $$

7 $$ {\text{Freundlich}}\;\left( {{\text{linear}}} \right):\log q_{e} = \log K_{F} + { }\frac{1}{n}\log C_{e} $$

where *q*_*e*_ (mg/g) and *q*_*m*_ (mg/g) denote equilibrium and maximum adsorption capacity, respectively. *K*_*L*_ (L/mg) is the Langmuir constant, where the correlation of the adsorbate with adsorbent surface. *K*_*F*_ [(mg/g)/(mg/L)^1/n^] represents the Freundlich constant associated with the adsorption capacity, while *n* indicates the adsorption intensity. These parameters of the Langmuir (*K*_*L*_ and *q*_*m*_) and Freundlich (*K*_*F*_ and *n*) adsorption isotherms are calculated using the plots of 1/*C*_*e*_ vs. 1/*q*_*e*_ and the log *C*_*e*_ vs. log *q*_*e*_, respectively. The adsorption isotherms were analyzed under similar conditions and processes as the Cs adsorption experiments; the ratio of sample to Cs-containing DI water, centrifugation, and filtration. The residual Cs concentration of the supernatant after the reaction was confirmed by ICP-MS.

### Cs desorption and reuse of adsorbent

The Cs desorption experiments were used to determine the adsorbent with the highest Cs removal efficiency, and were performed using 0.1 M HCl and NaOH to compare the desorption efficiency of acid and base solutions. These solutions were used under the following experimental conditions at RT: the ratio of adsorbent (g) to solution (mL) in the mixture, 1:100; reaction time, 10 min; agitation speed, 150 rpm. After the reaction, the supernatant was separated from the mixture via centrifugation at 6,000 rpm for 15 min, and then filtered using a 0.2 μm syringe filter. The Cs concentration in the filtered supernatant was analyzed by ICP-MS. To investigate the reusability of the adsorbent, the used adsorbent was washed twice with DI water, and then separated via centrifugation at 6,000 rpm for 15 min. The washed adsorbent was oven-dried at 60 °C.

## Conclusion

Opaline mudstone (OM) mainly consists of opal-CT (87.6%) with minor amounts of quartz and feldspar. OM had a high SSA of 67.58 m^2^/g, and a negative zeta potential value of − 6.66 mV. These properties make OM useful for Cs adsorption, and the adsorption capacity of OM can be improved using the acid- and base-activations as simple modification methods. The acid- and base-activations of OM increased the SSA from (67.58 to 101.62 and 107.36) m^2^/g, respectively. However, the Cs adsorption capacity of the acid-activated OM slightly decreased from (15.47 to 12.22) mg, while that of the base-activated OM doubled from (15.47 to 32.14) mg/g. The acid-activation of OM slightly increased the zeta potential from (− 6.66 to − 6.60) mV, and decreased pH from (4.24 to 3.07). The base-activation of OM largely decreased the zeta potential from (− 6.66 to − 16.67) mV, and increased pH from (4.24 to 9.92). The non- and acid-activated OM had low Cs removal efficiency of (44.9 and 34.6) %, respectively, as compared to that of the base-activated OM of 68.5%. In addition, the base-activated OM can be reused for at least four cycles with high Cs removal efficiency. From the aspect of the Cs adsorption mechanism, mineralogical properties and Cs adsorption results show that the base-activation of OM generates a pH-dependent negative charge by the deprotonation, which is advantageous for Cs adsorption through the electrostatic attraction. In the dynamic atomic behavior, the Cs cations adsorbed on the oxygen of Si‒O functional groups in opal-CT existed as a form of IS-type in the Stern layer, and partially hydrated. Consequently, opaline mudstone composed of opal-CT is expected to be used as an effective Cs adsorbent through base-activation as a simple surface modification method.

## Supplementary Information

Supplementary Information.
