# Supplementary material for: Effective cesium removal from Cs-containing water using chemically activated opaline mudstone mainly composed of opal-cristobalite/tridymite (opal-CT)
Source: Sci Rep. 2021 Jul 28;11:15362. doi: 10.1038/s41598-021-94832-y (PMC8319380; doi:10.1038/s41598-021-94832-y)
Supplement: Supplementary file 1 — Supplementary Information. [file 41598_2021_94832_MOESM1_ESM.pdf]

Supplementary Information

Effective cesium removal from Cs-containing water using chemically activated opaline mudstone mainly composed of opal-cristobalite/tridymite (opal–CT)

Sunki Kwon[1], Yumi Kim[1], Yul Roh[1*]

[1]*Department of Earth and Environmental Sciences, Chonnam national University, 77 Yongbong-ro, Buk-gu, Gwangju 61186, Republic of Korea*

*Corresponding author.

(Y. Roh) Tel.: +82-62-530-3458; E-mail address: rohy@jnu.ac.kr

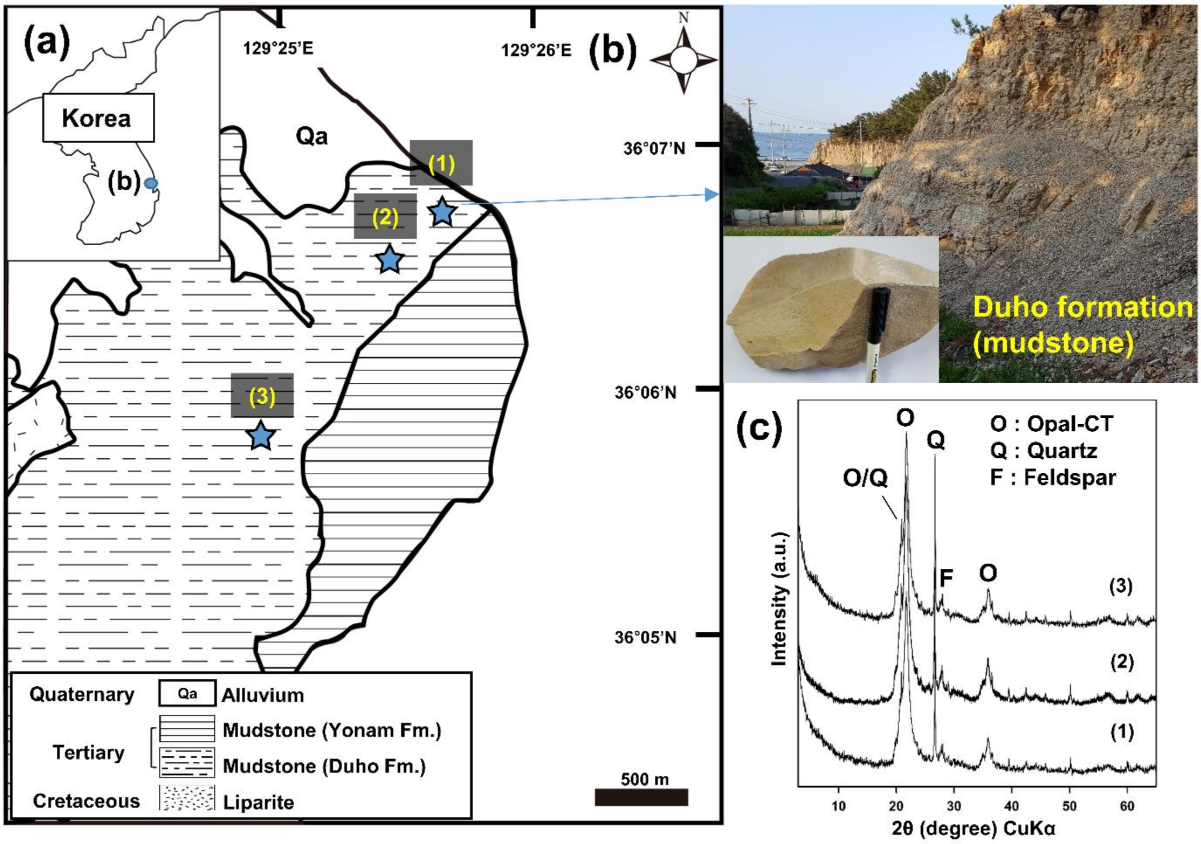

Figure S1. (a) Map of the Korean Peninsula, (b) simplified geological map, and (c) graphical representation of the XRD results of the mudstones of the Heunghae area in Pohang city, with photo of the Duho formation. The geological map was modified from the geological map of Korea Institute of Geoscience and Mineral Resources (KIGAM, https://data.kigam.re.kr/mgeo/map/main.do?process=geology_50k, April, 2021) using the Adobe Illustrator 2020.

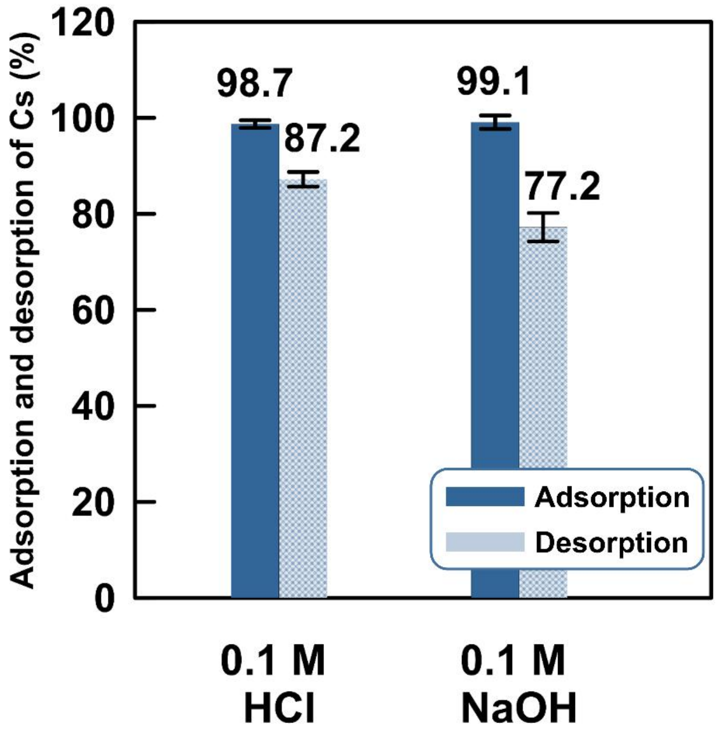

Figure S2. Adsorption of Cs from Cs-containing water using base-activated opaline mudstone and desorption of Cs from the Cs-adsorbed minerals by 0.1 M HCl and 0.1 M NaOH solutions.

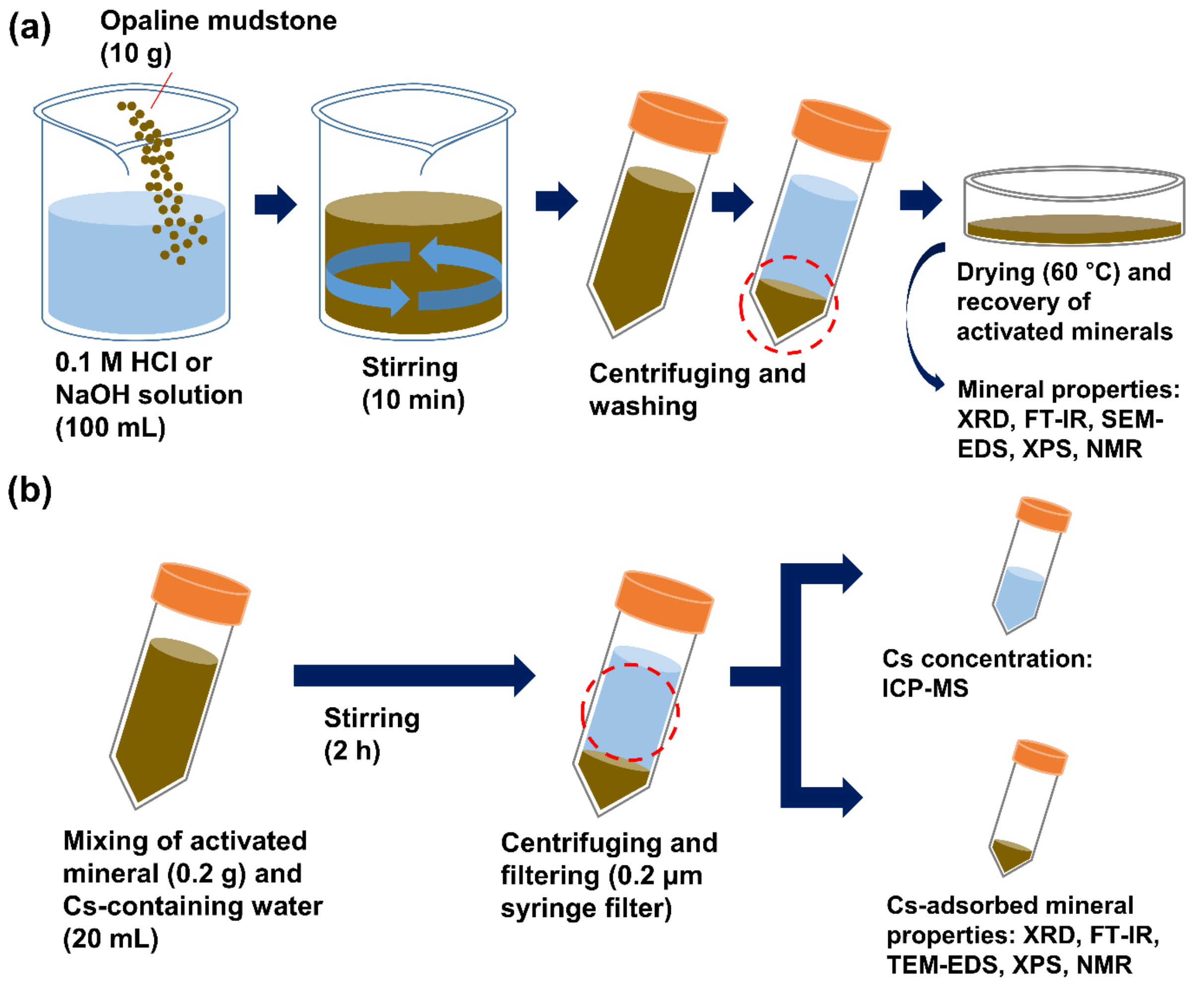

Figure S3. Schematic of the experimental processes for the (a) acid- and base-activation of opaline mudstone, and (b) Cs adsorption using chemically activated opaline mudstone.
